# Nfatc4 Deficiency Attenuates Ototoxicity by Suppressing Tnf-Mediated Hair Cell Apoptosis in the Mouse Cochlea

**DOI:** 10.3389/fimmu.2019.01660

**Published:** 2019-07-17

**Authors:** Yanping Zhang, Diyan Chen, Liping Zhao, Wen Li, Yusu Ni, Yan Chen, Huawei Li

**Affiliations:** ^1^State Key Laboratory of Medical Neurobiology, Department of Affiliated Eye and ENT Hospital, ENT Institute and Otorhinolaryngology, Institutes of Biomedical Sciences and the Institutes of Brain Science and the Collaborative Innovation Center for Brain Science, Fudan University, Shanghai, China; ^2^NHC Key Laboratory of Hearing Medicine, Fudan University, Shanghai, China; ^3^Shanghai Engineering Research Centre of Cochlear Implant, Shanghai, China

**Keywords:** Nfatc4, inflammation, inner ear, cochlear hair cells, cell apoptosis, hearing loss

## Abstract

The loss of sensory hair cells in the cochlea is the major cause of sensorineural hearing loss, and inflammatory processes and immune factors in response to cochlear damage have been shown to induce hair cell apoptosis. The expression and function of Nfatc4 in the cochlea remains unclear. In this study, we investigated the expression of Nfatc4 in the mouse cochlea and explored its function using *Nfatc4*^−/−^ mice. We first showed that *Nfatc4* was expressed in the cochlear hair cells. Cochlear hair cell development and hearing function were normal in *Nfatc4*^−/−^ mice, suggesting that Nfatc4 is not critical for cochlear development. We then showed that when the hair cells were challenged by ototoxic drugs Nfatc4 was activated and translocated from the cytoplasm to the nucleus, and this was accompanied by increased expression of *Tnf* and its downstream targets and subsequent hair cell apoptosis. Finally, we demonstrated that Nfatc4-deficient hair cells showed lower sensitivity to damage induced by ototoxic drugs and noise exposure compared to wild type controls. The Tnf-mediated apoptosis pathway was attenuated in Nfatc4-deficient cochlear epithelium, and this might be the reason for the reduced sensitivity of Nfatc4-deficient hair cells to injury. These findings suggest that the amelioration of inflammation-mediated hair cell apoptosis by inhibition of Nfatc4 activation might have significant therapeutic value in preventing ototoxic drug or noise exposure-induced sensorineural hearing loss.

## Introduction

Hearing loss is one of the most common sensory disorders in humans, and around 466 million people worldwide have disabling hearing loss. Sensorineural hearing loss might result from genetic causes, the use of ototoxic drugs, excessive noise exposure, and aging. Sensory hair cells in the cochlea detect sound and are responsible for converting mechanical signals into electrical signals, and aminoglycosides and excessive noise exposure can induce caspase-mediated apoptosis in hair cells and thus lead to hearing loss (1, 2).

Increasing evidence suggests that inflammatory processes play significant roles in the response to cochlear injury. For example, the expression levels of pro-inflammatory cytokines, chemokines, and cell adhesion molecules are increased in both acute and chronic noise-exposed mouse cochleae (3), and several clinical studies have shown that inflammation is a significant component of the mechanisms underlying presbycusis (4). Tumor necrosis factor (Tnf, also known as Tnf-α) is a central circulating factor that is essential for the systemic inflammatory mechanism. Tnf expression is increased in the mouse cochlea following acute and chronic noise exposure (3, 5), and exposure to Tnf can induce hair cell damage and apoptosis in cultured cochlear explants (6).

Nuclear factor of activated T cells 4 (*Nfatc4*), also known as *Nfat3*, encodes a member of the nuclear factor of activated T cells (NFAT) protein family, which are integral proteins in the development and function of the immune system. The activation of the NFAT family is controlled by calcineurin, a Ca^2+^-dependent phosphatase. Originally identified in T cells as inducers of cytokine gene expression (7), NFAT proteins have been shown to play various roles in cells outside of the immune system, including functions in cell development, differentiation, and adaptation (8) and in neuronal excitability (9). Nfatc4 is also involved in cell apoptosis in many tissues and organs. Nfatc4 is anti-apoptotic and mediates cell survival in some tissues, such as neurons (10), and the NMDAR-Nfatc4-BDNF pathway contributes to cell survival during cortical development (11). However, in other tissues, such as glioma cells and renal tubular cells, Nfatc4 mediates cell apoptosis. In glioma cells, Nfatc4 activation is a prerequisite for the induction of DOX-mediated apoptosis (12). In renal tubular cells, a carboplatin-mediated increase in reactive oxygen species (ROS) leads to Nfatc4 activation and cell apoptosis, and treatment with N-acetylcysteine, an antioxidant, blocks Nfatc4 activation and thus prevents cell apoptosis (13).

However, the expression and function of Nfatc4 in the cochlea remains unclear. In this study, we found that Nfatc4 is expressed in the cochlear hair cells and that when the hair cells were challenged by neomycin (an ototoxic drug) Nfatc4 translocated to the nucleus, enhanced the expression of *Tnf* and its downstream targets, and led to hair cell apoptosis. Furthermore, Nfatc4-deficient hair cells showed reduced sensitivity to ototoxic drugs and noise exposure compared to wild type (WT) controls. The Tnf-mediated apoptosis pathway was attenuated in Nfatc4-deficient hair cells, and this might be the reason for the reduced sensitivity of Nfatc4-deficient hair cells to injury.

## Materials and Methods

### Mice and Genotyping

Nfatc4 knockout mice were generated by Professor Gerald R. Crabtree (14) and provided by Dr. Yan-Ai Mei of Fudan University. As previously described (9, 14), *Nfatc4* knockout (*Nfatc4*^−/−^) mice were backcrossed 10–12 times onto C57BL/6 mice to obtain homozygous *Nfatc4* mice. *Nfatc4*^−/−^ and WT mice (littermates) were genotyped by PCR. The three primers used in genotyping were CCG GTG CAT CCC GGG TAA CCA ATC AGA GA; TCC TCA TCC TCG CAG CTT GCG GCC CC; and AGC GTT GGC TAC CCG TGA TAT TGC TGA AGA. The genotypes were identified by a 300 bp WT band and a 700 bp mutant band. Mice of both sexes were used. This study was carried out in strict accordance with the “Guiding Directive for Humane treatment of Laboratory Animals” issued by the Chinese National Ministry of Science and Technology in 2006. All experiments were approved by the Shanghai Medical Experimental Animal Administrative Committee (Permit Number: 2009-0082), and all efforts were made to minimize suffering and reduce the number of animals used.

### Experimental Protocol (*In vivo* Studies)

Hearing thresholds were measured in anesthetized postnatal day (P)30 or P33 mice by auditory brainstem response (ABR) analysis as described previously (2). The hearing thresholds were assessed at four frequencies (8, 16, 24, and 32 kHz) using a TDT system (Tucker Davies Technologies).

To explore the effect of Nfatc4 deficiency on sensitivity to noise exposure in cochlear hair cells, the hearing function of P30 *Nfatc4*^−/−^ and WT mice (littermates) was examined by ABR. Twenty-four hours after the ABR test, the mice were exposed to noise at 118 dB (8–16 kHz) for 2 h. At 2 days after noise exposure, hearing function was examined again by ABR, after which the mice were sacrificed and their cochleae were removed and fixed in 4% paraformaldehyde (PFA). After decalcification, the cochlear epithelium was prepared for morphological analysis.

### Experimental Protocol (*In vitro* Studies)

Cochlear sensory epithelium was dissected from P2 mice and cultured as previously reported (15). Neomycin (1 mM, Sigma) was added to the medium for 6 h to kill hair cells. After neomycin was removed, the tissues were cultured in serum-free medium for an additional 24 h before fixation. For the Tnf inhibition experiment, lenalidomide (Len) or etanercept (ETA) and neomycin were added simultaneously. For the inhibition of calcineurin/NFAT signaling, cyclosporin A (CsA) or tacrolimus (FK506) and neomycin were added simultaneously.

### Immunofluorescence and TUNEL Staining

Rabbit polyclonal anti-myosin VIIA (Myo7a) (Proteus Biosciences, 1:1,000 dilution), goat polyclonal anti-SRY (sex-determining region Y)-box 2 (Sox2) (Santa Cruz Biotechnology, 1:1,000 dilution), and mouse monoclonal anti-Nfatc4 (Santa Cruz Biotechnology, 1:200 dilution) were used. Corresponding donkey anti-rabbit, anti-goat, or anti-mouse secondary antibodies conjugated with Alexa Fluor 488, Alexa Fluor 568, or Alexa Fluor 647 (ThermoFisher Scientific, 1:500 dilution) were used. Immunofluorescence staining was performed as previously reported (2). A TUNEL Kit (Roche) was used to detect apoptotic cells according to the manufacturer's instructions. Specimens were examined by confocal fluorescence microscopy (Leica SP8).

### Real-Time PCR

Real-time PCR was performed on cochlear explants. A total of 2 μg total RNA was used for reverse transcription with Superscript III reverse transcriptase (Invitrogen), and real-time PCR was performed on an ABI 7500 real-time PCR system (Applied Biosystems) using the TB Green PrimeScript RT-PCR Kit (Takara). All primers were designed to flank individual exons and were tested by PCR. The optimized conditions were held constant for each sample to assure valid comparisons of the results. Primer sets were as follows: *Actb* (F) tct ttg cag ctc ctt cgt tg; (R) tcc ttc tga ccc att ccc ac; *Nfatc4* (F) tcg gag agg aaa agg agc c; (R) tgg tga gtg cat ccc tgg; *Tnf* (F) gcc tcc ctc tca tca gtt ct; (R) gca gcc ttg tcc ctt gaa g; *Casp8* (F) aac tgc gtt tcc tac cga ga; (R) cct tgt tcc tcc tgt cgt ct; *Casp3* (F) gag cag ctt tgt gtg tgt ga; (R) tgt ctc aat gcc aca gtc ca; *Casp9* (F) gga ccg tga caa act tga gc; *Casp9* (R) tct cca tca aag ccg tga cc. *Actb* was used as a housekeeping gene for control purposes. Each PCR reaction was carried out in triplicate, and the relative quantification of gene expression was analyzed using the 2^−ΔΔ*CT*^ method with *Actb* as the endogenous reference.

### Cell Counting and Statistical Analysis

To quantify the immunostaining-positive cells, nine separate segments along the entire cochlea were selected from the apex to the base (corresponding to approximately 4.0, 5.6, and 8.0 kHz in the apical turn; 11.3, 16.0, and 22.6 kHz in the middle turn; and 32.0, 45.2, and 64.0 kHz in the basal turn). Data are presented as the mean ± SD. Student's 2-tailed *t*-test was used to analyze the differences between two groups in Figures 4, 5. For the comparison of differences among three groups in Figure 6E, one-way ANOVA followed by Bonferroni *post-test* for multiple comparisons was used. For the comparison of differences among three or more groups in other figures, two-way ANOVA followed by Bonferroni *post-test* for multiple comparisons was used. *P* < 0.05 was considered significant, and the *p*-values are presented in the figures (^*^indicates *p* < 0.05, ^**^indicates *p* < 0.01, and ^***^indicates *p* < 0.001).

## Results

### Nfatc4 Was Expressed in the Cochlear Hair Cells

**Figure 1:**
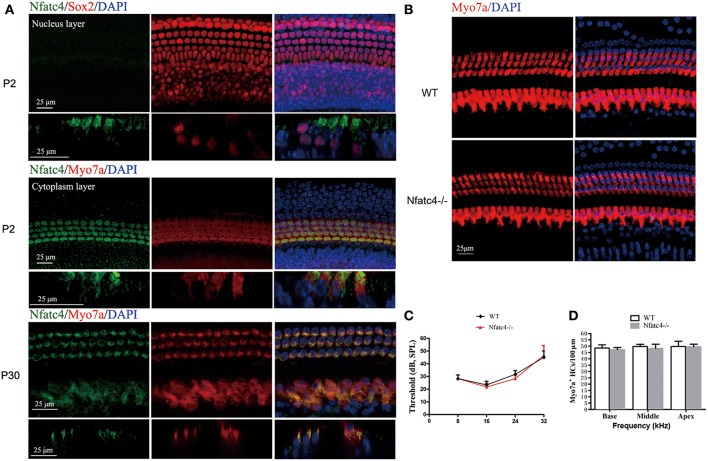
Nfatc4 was expressed in the cochlear hair cells, and *Nfatc4*^−/−^ mice showed normal cochlear development and hearing function. **(A)** Nfatc4 immunofluorescence staining in the cochlear hair cells (middle turns) of P2 and P30 WT mice. Myo7a and Sox2 were used as hair cell and supporting cell markers, respectively. **(B)** Myo7a immunofluorescence in cochlear hair cells (middle turn) of adult *Nfatc4*^−/−^ and WT mice. **(C)** The hearing thresholds of ABR measurement in the adult *Nfatc4*^−/−^ and WT mice. **(D)** The numbers of hair cells in *Nfatc4*^−/−^ and WT mice. Scale bars: 25 μm. *n* = 5.

Immunofluorescence staining with the anti-Nfatc4 antibody was performed to determine the extent of Nfatc4 expression in the mouse cochlea. Myo7a and Sox2 were used as hair cell and supporting cell markers, respectively. Double staining of Nfatc4 and Myo7a showed that Nfatc4 was expressed in hair cells in all three turns of the cochlea in both the neonatal and adult mice (Figure 1A). Double immunofluorescence staining of Nfatc4 and Sox2 showed that Nfatc4 was not expressed in cochlear supporting cells (Figure 1A).

### *Nfatc4*^−/−^ Mice Showed Normal Cochlear Development and Hearing Function

To explore the role of Nfatc4 in the development of cochlear hair cells and in hearing function, we used *Nfatc4*^−/−^ mice. In the adult stage, the morphological features and quantities of cochlear hair cells in the *Nfatc4*^−/−^ mice were similar to the WT controls (Figures 1B,D). The hearing function of adult *Nfatc4*^−/−^ mice was normal at 8, 16, 24, and 32 KHz as demonstrated by ABR measurement data (Figure 1C). These results suggested that *Nfatc4* is not a critical gene for cochlear development.

### Cochlear Hair Cells in *Nfatc4*^−/−^ Mice Showed Reduced Sensitivity to Aminoglycoside Antibiotic-Induced Ototoxicity

**Figure 2:**
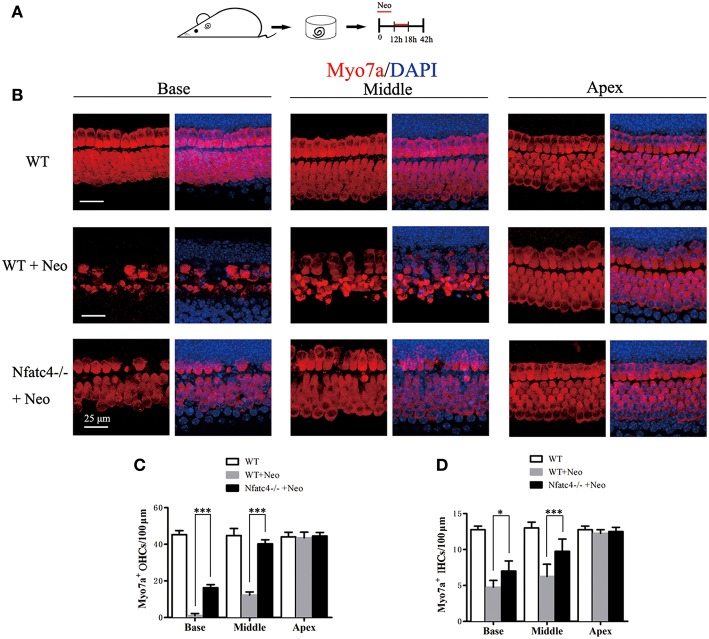
Cochlear hair cells in *Nfatc4*^−/−^ mice showed reduced sensitivity to aminoglycoside antibiotic-induced ototoxicity. **(A)** The diagram of the assay. Cochlear sensory epithelium samples from P2 *Nfatc4*^−/−^ and WT mice were dissected out and allowed to recover for 12 h. The samples were treated with 1 mM neomycin for 6 h, allowed to recover for 24 h, and then used for immunostaining. **(B)** The representative Myo7a immunofluorescence staining of sensory epithelium from *Nfatc4*^−/−^ and WT mice after neomycin treatment. **(C,D)** Quantification of inner hair cells (IHCs) and outer hair cells (OHCs). The numbers of IHCs and OHCs in the middle and basal turns were significantly greater in the cochlear epithelium from *Nfatc4*^−/−^ mice than in WT mice after neomycin treatment. Scale bar = 25 μm. *indicates *p* < 0.05 and ***indicates *p* < 0.001. *n* = 5.

We next investigated the role of Nfatc4 in hair cell injury using an aminoglycoside antibiotic injury model. In this model, the sensitivity of hair cells to ototoxic drug injury increased from the apical turn to the basal turn. Cultured cochlear epithelium tissues from *Nfatc4*^−/−^ and WT mice were treated with 1 mM neomycin for 6 h and harvested after an additional 24 h in culture (Figure 2A). Myo7a staining was then used to identify the remaining hair cells. In the WT control group with neomycin administration, the numbers of inner hair cells (IHCs) were 12.25 ± 0.5, 6.25 ± 1.71, and 4.75 ± 0.95 cells/100 μm in the apical, middle, and basal turns, respectively (Figures 2B,D), and the numbers of outer hair cells (OHCs) were 43.50 ± 3.11, 12.25 ± 1.71, and 1.00 ± 1.15 cells/100 μm in the apical, middle, and basal turns, respectively (Figures 2B,C). In the *Nfatc4*^−/−^ cochlear epithelium with neomycin administration, more hair cells were observed, and there were 12.5 ± 0.58, 9.75 ± 1.71, and 7.00 ± 1.41 cochlear IHCs/100 μm and 44.50 ± 1.91, 40.25 ± 2.22, and 16.25 ± 1.71 cochlear OHCs/100 μm in the apical, middle, and basal turns, respectively (Figures 2B–D). The numbers of hair cells in the middle and basal turns were significantly greater in the cochlear epithelium from *Nfatc4*^−/−^ mice compared with control mice, indicating that *Nfatc4*^−/−^ cochlear hair cells were less sensitive to aminoglycoside antibiotic-induced injury.

### Nfatc4 Deficiency Protected Against Noise-Induced Hearing Loss and Hair Cell Loss

**Figure 3:**
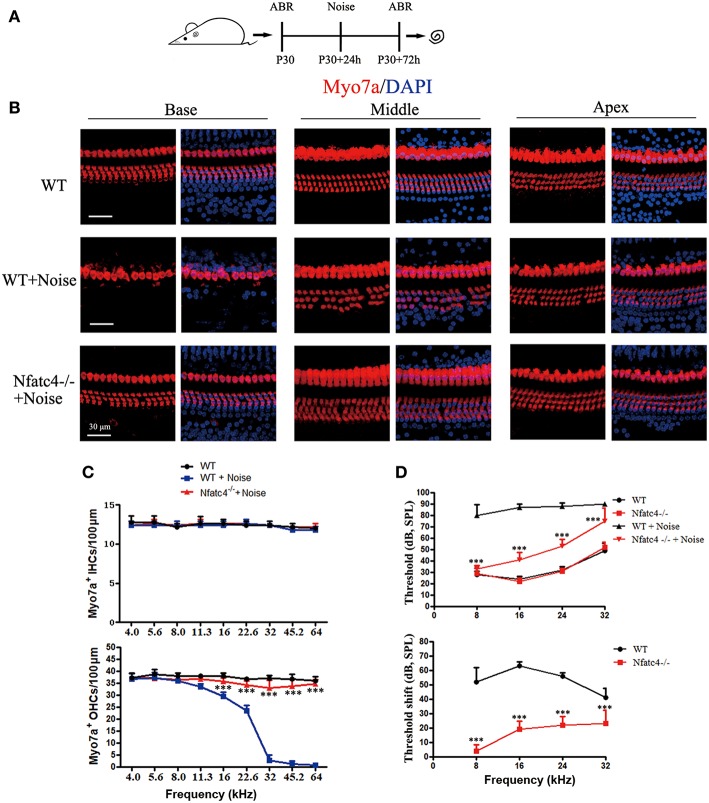
Nfatc4-deficient mice displayed enhanced resistance to hearing loss induced by noise exposure. **(A)** The diagram of the assay for **B–D**. P30 *Nfatc4*^−/−^ and WT mice (littermates) were examined by ABR, then 24 h later exposed to 118 dB noise (8–16 kHz) for 2 h. At 2 days after noise exposure, hearing function was examined again by ABR and the mouse cochleae were harvested. **(B)** The representative Myo7a immunofluorescence staining of sensory epithelium from *Nfatc4*^−/−^ and WT mice after noise exposure. **(C)** Quantification of inner hair cells (IHCs) and outer hair cells (OHCs). The numbers of OHCs in the middle and basal turns were significantly greater in the cochlear epithelium from *Nfatc4*^−/−^ mice than in WT mice after noise exposure. **(D)** Pure-tone ABR thresholds and threshold shift of *Nfatc4*^−/−^ mice and WT mice after noise exposure. Scale bar = 30 μm. *n* = 5. ***P < 0.001 vs. the WT+Noise group.

To further explore the role of Nfatc4 in the adult cochlea, we used a noise exposure hair cell injury model. In this model, the sensitivity of hair cells to noise injury depends on the noise frequency, intensity, and duration. In this experiment, adult mice were exposed to 118 dB noise (8–16 kHz) for 2 h, which leads to OHC loss in the apical-middle, middle, and basal turns and thus results in permanent hearing loss (16). Hearing function was examined by ABR at 2 days after noise exposure, and then the cochleae were harvested (Figure 3A). In the control group, noise exposure led to significant OHC loss in the cochlear turn at 11.3, 16.0, 22.6, 32.0, 45.2, and 64.0 kHz, which was consistent with results from a previous study (16). However, in *Nfatc4*^−/−^ mice the noise-induced loss of OHCs was significantly decreased in the middle and basal turns (at 16.0, 22.6, 32.0, 45.2, and 64.0 kHz) compared with control mice (Figures 3B,C), indicating that adult *Nfatc4-*deficient cochlear hair cells were more tolerant of noise.

At 2 days after noise exposure, the WT mice had significant hearing loss, as demonstrated by the increased ABR thresholds at 8, 16, 24, and 32 kHz (Figure 3D). In Nfatc4-deficient mice, the ABR threshold shifts were significantly lower at all frequencies compared with the control mice after noise exposure (Figure 3D), suggesting that Nfatc4 deficiency protects against noise-induced hearing loss *in vivo*.

### Neomycin Treatment Triggered Nfatc4 Nuclear Translocation, Tnf Pathway Activation, and Hair Cell Apoptosis

**Figure 4:**
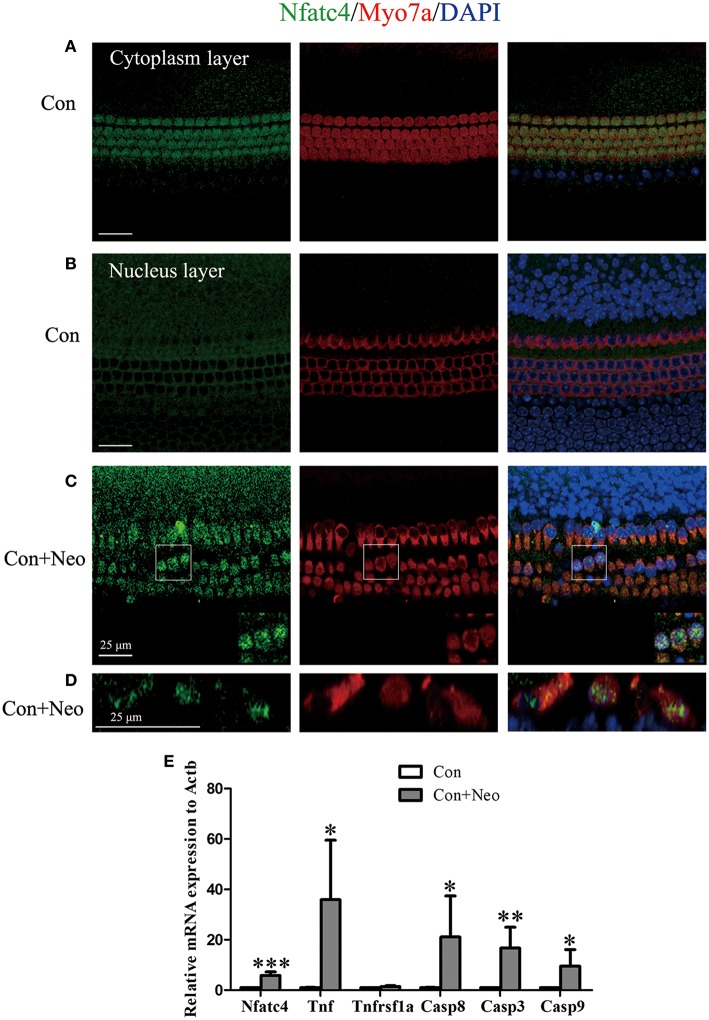
Neomycin treatment triggered Nfatc4 nuclear translocation, Tnf pathway activation, and hair cell apoptosis. **(A–C)** Immunofluorescence staining showed the translocation of Nfatc4 from the cytoplasm to the nucleus. **(D)** The cross-sectional view of Nfatc4 staining in neomycin-treated cochlear epithelium. **(E)** qPCR analysis showed that *Nfatc4, Tnf*, *Casp3, Casp9*, and *Casp8* mRNA expression increased in the cochlear epithelium after neomycin damage. Scale bar = 25 μm. *indicates *p* < 0.05, **indicates *p* < 0.01, and ***indicates *p* < 0.001 vs. the Con group. *n* = 5.

Previous studies have reported Nfatc4 nuclear translocation in response to stress, such as methamphetamine-induced neuronal injury (17). In this study, we also observed Nfatc4 translocation from the cytoplasm to the nucleus when the hair cells were challenged by neomycin exposure. Under normal conditions, Nfatc4 was located in the cytoplasm of hair cells (Figures 4A,B). After the neomycin treatment, Nfatc4 was translocated to the hair cell nuclei (Figure 4C). Neomycin-induced Nfatc4 nuclear translocation occurred in nearly all the hair cells across the cochlear length as shown in Figure 4D. In previous studies, chromatin immunoprecipitation assays confirmed that the recruitment of Nfatc4 to the *Tnf* promoter was required in ultraviolet radiation-induced cell death (18), indicating that Tnf is a target of Nfatc4. In this study, we explored the expression of *Tnf* and its downstream signaling in cell apoptosis. As Figure 4E shows, after neomycin treatment the expression levels of *Tnf*, *Casp8*, and *Casp3* were significantly increased, suggesting that the Tnf pathway might play roles in neomycin-induced hair cell death. In addition, the mRNA expression of *Nfatc4* was increased after neomycin treatment (Figure 4E).

### The Tnf-Mediated Cell Apoptosis Pathway Was Inhibited in Nfatc4-Deficient Cochleae After Exposure to Aminoglycoside Antibiotics

**Figure 5:**
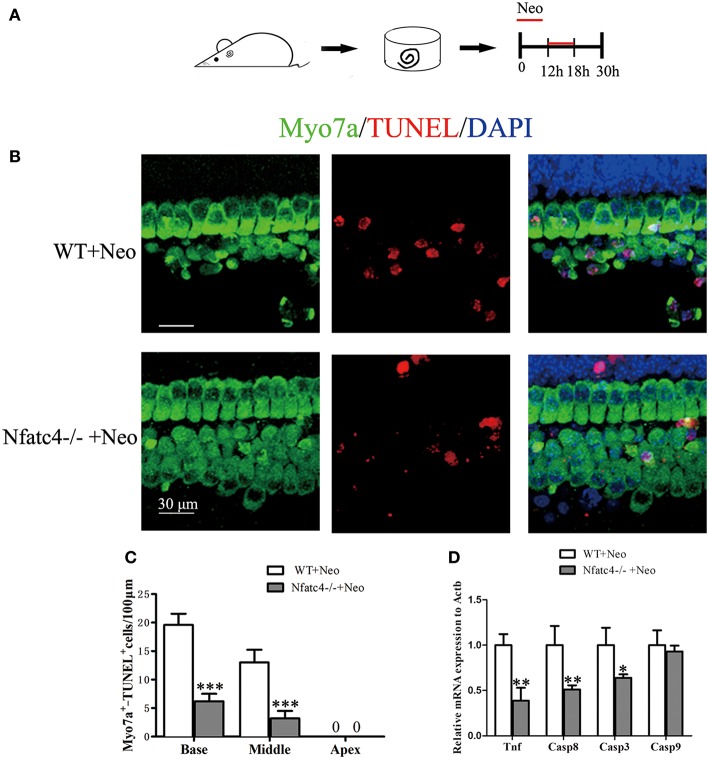
The Tnf-mediated cell apoptosis pathway was inhibited and hair cell apoptosis was decreased in Nfatc4-deficient cochleae after neomycin exposure. **(A)** The diagram of the assay for **(B–D)**. Cochlear sensory epithelium samples from P2 *Nfatc4*^−/−^ and WT mice were dissected out and allowed to recover for 12 h. The samples were treated with 1 mM neomycin for 6 h, allowed to recover for 12 h, and then used for TUNEL staining. **(B)** The representative Myo7a and TUNEL staining of sensory epithelium from *Nfatc4*^−/−^ and WT mice after neomycin treatment. **(C)** The numbers of Myo7a^+^/TUNEL^+^ cells in the middle and basal turns were significantly decreased in the cochlear epithelium from *Nfatc4*^−/−^ mice compared to WT mice after neomycin treatment. **(D)** qPCR analysis showed that the expression levels of *Tnf*, *Casp3*, and *Casp8* were decreased in Nfatc4-deficient cochlear epithelium after neomycin damage. Scale bar = 30 μm. *indicates *p* < 0.05, **indicates *p* < 0.01, and ***indicates *p* < 0.001 vs. the WT+Neo group. *n* = 5.

TUNEL staining was used to detect hair cell apoptosis (Figure 5A). In the WT control group, there were 13 ± 1.00 TUNEL+/Myo7a+ cells/100 μm in the middle turn of the cochlear epithelium after neomycin treatment. Consistent with the above results, the number of apoptotic hair cells was significantly decreased in the *Nfatc4*^−/−^ cochlear epithelium to 3.2 ± 1.30 TUNEL+/Myo7a+ cells/100 μm in the middle turn (*p* < 0.001, Figures 5B,C). In the Nfatc4-deficient cochlear epithelium, the expression levels of *Tnf* and its downstream cascade (*Casp8* and *Casp3*) were significantly lower compared with the WT group after neomycin treatment (Figure 5D), indicating that the neomycin-induced and Tnf-mediated extrinsic apoptosis pathway was attenuated by Nfatc4 deficiency. However, the expression levels of *Casp9* were not significantly changed by Nfatc4 deficiency (Figure 5D), suggesting that the neomycin-induced intrinsic apoptosis pathway was not inhibited by Nfatc4 deficiency. These results indicated that Nfatc4 deficiency attenuates the hair cell apoptosis induced by neomycin injury, and this might be due to inhibition of the Tnf pathway.

### Inhibition of Tnf Production Protected Cochlear Hair Cells Against Neomycin-Induced Damage

**Figure 6:**
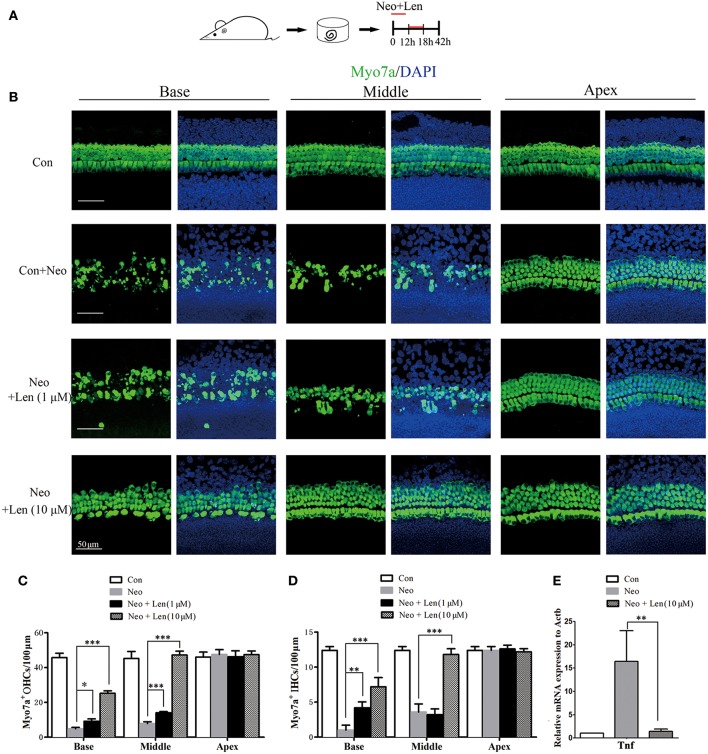
Inhibition of Tnf production protected cochlear hair cells against neomycin-induced damage. **(A)** The diagram of the assay for **(B–D)**. Cochlear sensory epithelium samples from P2 WT mice were dissected out and allowed to recover for 12 h. The samples were treated with 1 mM neomycin and Len (1 or 10 μM) for 6 h, allowed to recover for 24 h, and then used for immunostaining. **(B)** Representative Myo7a immunofluorescence staining after drug treatment (neomycin alone or combined with Len). **(C,D)** Len treatment protected against neomycin-induced hair cell loss. **(E)** qPCR analysis showed that Len significantly decreased the mRNA expression of *Tnf* in neomycin-injured cochlear epithelium. Scale bar = 50 μm. *indicates *p* < 0.05, **indicates *p* < 0.01, and ***indicates *p* < 0.001. *n* = 5.

To further investigate the role of the Tnf pathway in hair cell damage, we used Len, an inhibitor of Tnf production (19, 20). Len (1 or 10 μM) and 1 mM neomycin were added simultaneously to the medium for 6 h, and cochlear epithelia were harvested after an additional 24 h (Figure 6A). qPCR was performed to confirm the inhibitory effect of Len on Tnf production, and the results showed that Len significantly decreased the mRNA expression of *Tnf* in neomycin-injured cochlear epithelium (Figure 6E). In the neomycin-treated group, there were 3.60 ± 1.14 and 1.00 ± 0.71 IHCs/100 μm and 7.80 ± 1.10 and 4.80 ± 0.83 OHCs/100 μm in the middle and basal turns, respectively (Figures 6B–D). In the neomycin combined with 1 μM Len-treated group, there were 3.20 ± 0.84 and 4.20 ± 0.84 IHCs/100 μm and 14.00 ± 0.71 and 8.20 ± 0.83 OHCs/100 μm in the middle and basal turns, respectively (Figures 6B–D). In the neomycin combined with 10 μM Len-treated group, there were 11.80 ± 0.83 and 7.20 ± 1.30 IHCs/100 μm and 47.20 ± 2.28 and 25.20 ± 1.48 OHCs/100 μm in the middle and basal turns, respectively (Figures 6B–D). The number of hair cells was significantly greater in the neomycin combined with Len-treated cochlear epithelium compared to the neomycin-treated group, indicating that inhibition of Tnf production significantly reduced neomycin-induced hair cell loss.

To further confirm the effect of inhibiting Tnf on neomycin-induced ototoxicity, etanercept (ETA, 1 or 10 μg/ml), a soluble Tnf decoy receptor drug that binds to Tnf and inhibits its interaction with endogenous Tnf receptors (21, 22), was used to block the Tnf pathway. Significantly more hair cells were observed in the neomycin combined with ETA (10 μg/ml)-treated cochlear epithelium compared to the neomycin-treated group in the middle and basal turns (Figure S1, *p* < 0.05), indicating that ETA administration significantly reduced neomycin-induced hair cell loss and further confirmed the protective role of Tnf inhibition in cochlear hair cells.

## Discussion

Some genes related to the immune system have been shown to play diverse roles in cochlear development, hearing function, and hearing protection. For example, macrophage migration inhibitory factor (Mif), an inflammatory cytokine, acts as a neurotrophin in the developing inner ear (23). *Mif* knockout mice are hearing impaired and demonstrate fewer sensory hair cells and altered innervation of the organ of Corti. A missense mutation of *NLRP3*, which encodes the NLRP3 inflammasome, causes autosomal-dominant sensorineural hearing loss accompanied by autoinflammatory signs and symptoms (24).

Aging (4, 25), cisplatin treatment (26–28), and noise injury (1) have all been shown to activate the immune system and to lead to the production of inflammatory mediators in the cochlea, and these factors can have diverse actions in the inner ear. For example, activation of Toll-like receptor 4 activates the innate immune system and promotes sensory cell degeneration and cochlear dysfunction after acoustic injury (1), while activation of the adenosine A1 receptor protects against cisplatin ototoxicity in the cochlea by suppressing the NOX3/STAT1 inflammatory pathway (27). In addition, controlling inflammation by inhibition of STAT1 via siRNA protects against cisplatin-induced hair cell apoptosis (26).

In the embryonic brain, NFAT signaling is required to stimulate axon outgrowth (29), and Nfatc4 inhibits the expression of growth-associated protein 43 during neuronal maturation (30). In addition, the NMDAR-Nfatc4-BDNF pathway contributes to cell survival during the development of cortical neurons (11). In this study, we showed that Nfatc4 was expressed in mouse cochlear hair cells, but deficiency of Nfatc4 did not result in hair cell loss or in hearing malfunction (Figure 1), suggesting that Nfatc4 is not involved in the development of the auditory system.

The overproduction of ROS contributes to aminoglycoside-induced and noise exposure-induced hair cell death and associated hearing loss (2, 15, 31), and it has been reported that ROS activate Nfatc4 and lead to apoptosis in renal tubular cells (32). In this study, we showed that neomycin treatment activated Nfatc4 in cochlear hair cells, as demonstrated by the nuclear translocation of the Nfatc4 protein and the increased mRNA expression levels of *Nfatc4* (Figure 4), suggesting that Nfatc4 activation is involved in hair cell apoptosis. Nfatc4-deficient cochlear hair cells showed lower sensitivity to neomycin treatment and noise exposure (Figures 2, 3), again indicating that Nfatc4 activation mediates hair cell death in response to injury.

It was previously reported that Nfatc4 works upstream of Tnf-induced apoptosis of glioma cells (12), and the recruitment of Nfatc4 to the *Tnf* promoter has been shown to be required in ultraviolet radiation-induced cell death in mouse embryonic fibroblasts (18). The noise exposure-mediated increase in Tnf expression has also been reported previously (3, 5). In this study, we found that neomycin treatment resulted in the activation of the Tnf-induced cell apoptosis pathway, as demonstrated by increased mRNA levels of *Tnf*, *Casp8*, and *Casp3* (Figure 4). In addition, the inhibition of Tnf production with Len or blocking Tnf with ETA significantly protected hair cells against neomycin-induced hair cell loss, suggesting that the Tnf pathway mediated hair cell death in response to neomycin injury (Figure 6 and Figure S1). Moreover, we found that the mRNA levels of *Tnf*, *Casp8*, and *Casp3* were significantly decreased in Nfatc4-deficient cochlear epithelium compared with controls (Figure 5), indicating that Nfatc4 deficiency inhibited the neomycin-induced activation of the Tnf pathway and subsequent apoptosis. Although a previous study reported that Tnf-α treatment results in NFAT nuclear translocation via activation of calcineurin in the neuroblastoma cells (33), in our experiments Nfatc4 nuclear translocation was not blocked by Tnf inhibition using Len (Figure S3), suggesting that Nfatc4 works upstream of Tnf in neomycin-induced hair cell apoptosis. Taken together, these results suggest that the protective role of Nfatc4 deficiency in cochlear hair cells might be attributed to the inhibition of Tnf-mediated apoptosis.

In other tissues, including the brain, the mechanism(s) of Nfatc4 activation after injury mainly focus on calcineurin/NFAT signaling. The calcineurin/NFAT signaling inhibitors cyclosporin A (CsA) and tacrolimus (FK506) have been used in studies on the role of NFAT signaling in the survival of leukemia cells (34), T cell activation (35), and myeloid haematopoiesis (36). We used these two inhibitors to test whether they could protect cochlear hair cells against ototoxic drugs. We found that CsA administration had no obvious effect on the survival of cochlear hair cells (Figure S2). Neomycin-induced hair cell damage was only slightly attenuated by FK506 administration (Figure S2), and the protective effect of FK506 was far less than that of Nfatc4 deficiency or Len (a Tnf inhibitor). The possible role of calcineurin in the Nfatc4 activation in cochlear hair cells remains unclear.

In summary, this study reports the expression and function of Nfatc4 in cochlear hair cells. Although Nfatc4 does not appear to be involved in the development of cochlear hair cells or hearing function, Nfatc4 activation appears to mediate hair cell apoptosis induced by aminoglycoside drugs and noise exposure. Moreover, we have shown that Tnf-mediated apoptosis is involved in hair cell death and that Nfatc4 deficiency suppresses the activation of the Tnf pathway, thus protecting hair cells against caspase-mediated apoptosis after neomycin injury. These results suggest that Nfatc4 inhibition might be a new therapeutic target for the prevention of aminoglycoside and noise-induced hearing loss.

## Data Availability

The raw data supporting the conclusions of this manuscript will be made available by the authors, without undue reservation, to any qualified researcher.

## Ethics Statement

This study was carried out in strict accordance with the Guiding Directive for Humane treatment of Laboratory Animals issued by the Chinese National Ministry of Science and Technology in 2006. All experiments were approved by the Shanghai Medical Experimental Animal Administrative Committee (Permit Number: 2009-0082), and all efforts were made to minimize suffering and reduce the number of animals used.

## Author Contributions

HL, YC, and YN conceived and designed the experiments. YZ, DC, WL, and LZ performed and analyzed the experiments. YZ, YC, and HL wrote the paper.

### Conflict of Interest Statement

The authors declare that the research was conducted in the absence of any commercial or financial relationships that could be construed as a potential conflict of interest.

## Supplementary Material

Figure S1 Etanercept treatment protected cochlear hair cells against neomycin-induced damage. **(A)** The diagram of the assay for **(B–D)**. Cochlear sensory epithelium samples from P2 WT mice were dissected out and allowed to recover for 12 h. The samples were treated with 1 mM neomycin and etanercept (ETA, 1 μg/ml or 10 μg/ml) for 6 h, allowed to recover for 24 h, and then used for immunostaining. **(B)** The representative Myo7a immunofluorescence staining after drug treatment (neomycin alone or combined with ETA). **(C,D)** ETA treatment at 10 μg/ml protected against neomycin-induced hair cell loss. Scale bar = 20 μm. ^***^indicates *p* < 0.001. *n* = 5.

Figure S2 The effects of calcineurin/NFAT signaling inhibitors on the survival of cochlear hair cells against neomycin-induced damage. **(A)** The diagram of the assay for **(B,C)**. Cochlear sensory epithelium samples from P2 WT mice were dissected out and allowed to recover for 12 h. The samples were treated with 1 mM neomycin and CsA/FK506 for 6 h, allowed to recover for 24 h, and then used for immunostaining. **(B)** The representative Myo7a immunofluorescence staining after drug treatment. **(C)** Quantification of the number of HCs. Scale bar = 20 μm. ^*^indicates *p* < 0.05. *n* = 5.

Figure S3 Immunofluorescence staining of Nfatc4 in neomycin combined with Len treated cochlear epithelium. Scale bar = 10 μm.

## Abbreviations

Tnf: tumor necrosis factor
Nfatc4: Nuclear factor of activated T cells 4
NMDAR: N-methyl-D-aspartate receptor
BDNF: brain derived necrosis factor
ROS: reactive oxygen species
WT: wild type
ABR: auditory brainstem response
TDT system: Tucker Davies Technologies
PFA: 4% paraformaldehyde
Myo7a: myosin VIIA
IHCs: inner hair cells
OHCs: outer hair cells
Casp8: caspase 8
Casp9: caspase 9
Casp3: caspase 3
Len: Lenalidomide
ETA: Etanercept
Mif: migration inhibitory factor
TLR 4: Toll-like receptor 4
NOX3: NADPH oxidase 3
STAT1: signal transducers and activators of transcription 1
CsA: Cyclosporin A
FK506: Tacrolimus.

## References

[1] Vethanayagam RR Yang W Dong Y Hu BH. Toll-like receptor 4 modulates the cochlear immune response to acoustic injury. Cell Death Dis. (2016) 7:e2245. 10.1038/cddis.2016.156 27253409 PMC5143385

[2] Chen Y Li L Ni W Zhang Y Sun S Miao D. Bmi1 regulates auditory hair cell survival by maintaining redox balance. Cell Death Dis. (2015) 6:e1605. 10.1038/cddis.2014.549 25611380 PMC4669747

[3] Tan WJT Thorne PR Vlajkovic SM. Characterisation of cochlear inflammation in mice following acute and chronic noise exposure. Histochem Cell Biol. (2016) 146:219–30. 10.1007/s00418-016-1436-5 27109494

[4] Watson N Ding B Zhu X Frisina RD. Chronic inflammation - inflammaging - in the ageing cochlea: a novel target for future presbycusis therapy. Ageing Res Rev. (2017) 40:142–8. 10.1016/j.arr.2017.10.002 29017893 PMC5675822

[5] Fuentes-Santamaria V Alvarado JC Melgar-Rojas P Gabaldon-Ull MC Miller JM Juiz JM. The role of glia in the peripheral and central auditory system following noise overexposure: contribution of TNF-alpha and IL-1beta to the pathogenesis of hearing loss. Front Neuroanat. (2017) 11:9. 10.3389/fnana.2017.00009 28280462 PMC5322242

[6] Wu Q Wang GP Xie J Guo JY Gong SS. Tumor necrosis factor-alpha-induced ototoxicity in mouse cochlear organotypic culture. PLoS ONE. (2015) 10:e0127703. 10.1371/journal.pone.0127703 26000970 PMC4441368

[7] Rao A Luo C Hogan PG. Transcription factors of the NFAT family: regulation and function. Annu Rev Immunol. (1997) 15:707–47. 10.1146/annurev.immunol.15.1.707 9143705

[8] Horsley V Pavlath GK. NFAT: ubiquitous regulator of cell differentiation and adaptation. J Cell Biol. (2002) 156:771–4. 10.1083/jcb.200111073 11877454 PMC2173310

[9] Yao JJ Zhao QR Liu DD Chow CW Mei YA. Neuritin up-regulates Kv4.2 alpha-subunit of potassium channel expression and affects neuronal excitability by regulating the calcium-calcineurin-NFATc4 signaling pathway. J Biol Chem. (2016) 291:17369–81. 10.1074/jbc.M115.708883 27307045 PMC5016134

[10] Benedito AB Lehtinen M Massol R Lopes UG Kirchhausen T Rao A. The transcription factor NFAT3 mediates neuronal survival. J Biol Chem. (2005) 280:2818–25. 10.1074/jbc.M408741200 15537643

[11] Vashishta A Habas A Pruunsild P Zheng JJ Timmusk T Hetman M. Nuclear factor of activated T-cells isoform c4 (NFATc4/NFAT3) as a mediator of antiapoptotic transcription in NMDA receptor-stimulated cortical neurons. J Neurosci. (2009) 29:15331–40. 10.1523/Jneurosci.4873-09.2009 19955386 PMC2836809

[12] Gopinath S Vanamala SK Gujrati M Klopfenstein JD Dinh DH Rao JS. Doxorubicin-mediated apoptosis in glioma cells requires NFAT3. Cell Mol Life Sci. (2009) 66:3967–78. 10.1007/s00018-009-0157-5 19784808 PMC2809824

[13] Lin H Sue YM Chou Y Cheng CF Chang CC Li HF. Activation of a nuclear factor of activated T-lymphocyte-3 (NFAT3) by oxidative stress in carboplatin-mediated renal apoptosis. Br J Pharmacol. (2010) 161:1661–76. 10.1111/j.1476-5381.2010.00989.x 20718735 PMC3010574

[14] Graef IA Chen F Chen L Kuo A Crabtree GR Signals transduced by Ca^2+^/calcineurin and NFATc3/c4 pattern the developing vasculature. Cell. (2001) 105:863–75. 10.1016/s0092-8674(01)00396-8 11439183

[15] Liu L Chen Y Qi J Zhang Y He Y Ni W. Wnt activation protects against neomycin-induced hair cell damage in the mouse cochlea. Cell Death Dis. (2016) 7:e2136. 10.1038/cddis.2016.35 26962686 PMC4823936

[16] Hirose K Liberman MC. Lateral wall histopathology and endocochlear potential in the noise-damaged mouse cochlea. J Assoc Res Otolaryngol. (2003) 4:339–52. 10.1007/s10162-002-3036-4 14690052 PMC1805786

[17] Jayanthi S Deng XL Ladenheim B McCoy MT Cluster A Cai NS. Calcineurin/NFAT-induced up-regulation of the Fas ligand/Fas death pathway is involved in methamphetamine-induced neuronal apoptosis. Proc Natl Acad Sci USA. (2005) 102:868–73. 10.1073/pnas.0404990102 15644446 PMC545515

[18] Song L Li J Ye J Yu G Ding J Zhang D. p85alpha acts as a novel signal transducer for mediation of cellular apoptotic response to UV radiation. Mol Cell Biol. (2007) 27:2713–31. 10.1128/MCB.00657-06 17242187 PMC1899908

[19] Wang Y Xu J Zhang X Wang C Huang Y Dai K. TNF-alpha-induced LRG1 promotes angiogenesis and mesenchymal stem cell migration in the subchondral bone during osteoarthritis. Cell Death Dis. (2017) 8:e2715. 10.1038/cddis.2017.129 28358372 PMC5386532

[20] Lapalombella R Andritsos L Liu Q May SE Browning R Pham LV. Lenalidomide treatment promotes CD154 expression on CLL cells and enhances production of antibodies by normal B cells through a PI3-kinase-dependent pathway. Blood. (2010) 115:2619–29. 10.1182/blood-2009-09-242438 19965642 PMC2852364

[21] Lee JK McCoy MK Harms AS Ruhn KA Gold SJ Tansey MG. Regulator of G-protein signaling 10 promotes dopaminergic neuron survival via regulation of the microglial inflammatory response. J Neurosci. (2008) 28:8517–28. 10.1523/JNEUROSCI.1806-08.2008 18716210 PMC2739568

[22] Herrmann M Anders S Straub RH Jenei-Lanzl Z. TNF inhibits catecholamine production from induced sympathetic neuron-like cells in rheumatoid arthritis and osteoarthritis *in vitro*. Sci Rep. (2018) 8:9645. 10.1038/s41598-018-27927-8 29941879 PMC6018168

[23] Bank LM Bianchi LM Ebisu F Lerman-Sinkoff D Smiley EC Shen YC. Macrophage migration inhibitory factor acts as a neurotrophin in the developing inner ear. Development. (2012) 139:4666–74. 10.1242/dev.066647 23172918 PMC3509728

[24] Nakanishi H Kawashima Y Kurima K Chae JJ Ross AM Pinto-Patarroyo G. NLRP3 mutation and cochlear autoinflammation cause syndromic and nonsyndromic hearing loss DFNA34 responsive to anakinra therapy. Proc Natl Acad Sci USA. (2017) 114:E7766–75. 10.1073/pnas.1702946114 28847925 PMC5604003

[25] Kao SY Soares VY Kristiansen AG Stankovic KM. Activation of TRAIL-DR5 pathway promotes sensorineural degeneration in the inner ear. Aging Cell. (2016) 15:301–8. 10.1111/acel.12437 26791792 PMC4783338

[26] Kaur T Mukherjea D Sheehan K Jajoo S Rybak LP Ramkumar V. Short interfering RNA against STAT1 attenuates cisplatin-induced ototoxicity in the rat by suppressing inflammation. Cell Death Dis. (2011) 2:63. 10.1038/cddis.2011.63 21776018 PMC3199718

[27] Kaur T Borse V Sheth S Sheehan K Ghosh S Tupal S. Adenosine A1 receptor protects against cisplatin ototoxicity by suppressing the NOX3/STAT1 inflammatory pathway in the cochlea. J Neurosci. (2016) 36:3962–77. 10.1523/JNEUROSCI.3111-15.2016 27053204 PMC4821909

[28] Borse V Al Aameri RFH Sheehan K Sheth S Kaur T Mukherjea D. Epigallocatechin-3-gallate, a prototypic chemopreventative agent for protection against cisplatin-based ototoxicity. Cell Death Dis. (2017) 8:e2921. 10.1038/cddis.2017.314 28703809 PMC5550861

[29] Graef IA Wang F Charron F Chen L Neilson J Tessier-Lavigne M. Neurotrophins and netrins require calcineurin/NFAT signaling to stimulate outgrowth of embryonic axons. Cell. (2003) 113:657–70. 10.1016/s0092-8674(03)00390-8 12787506

[30] Nguyen T Lindner R Tedeschi A Forsberg K Green A Wuttke A. NFAT-3 is a transcriptional repressor of the growth-associated protein 43 during neuronal maturation. J Biol Chem. (2009) 284:18816–23. 10.1074/jbc.M109.015719 19443652 PMC2707217

[31] Park JS Jou I Park SM. Attenuation of noise-induced hearing loss using methylene blue. Cell Death Dis. (2014) 5:170. 10.1038/Cddis.2014.170 24763057 PMC4001318

[32] Sue YM Chou HC Chang CC Yang NJ Chou Y Juan SH L-Carnitine protects against carboplatin-mediated renal injury: AMPK- and PPAR alpha-dependent inactivation of NFAT3. PLoS ONE. (2014) 9:0104079 10.1371/journal.pone.0104079 PMC4121315 25090113

[33] Alvarez S Blanco A Fresno M Munoz-Fernandez MA. TNF-alpha contributes to caspase-3 independent apoptosis in neuroblastoma cells: role of NFAT. PLoS ONE. (2011) 6:e16100. 10.1371/journal.pone.0016100 21298033 PMC3029262

[34] Gregory MA Phang TL Neviani P Alvarez-Calderon F Eide CA O'Hare T. Wnt/Ca^2+^/NFAT signaling maintains survival of Ph^+^ leukemia cells upon inhibition of Bcr-Abl. Cancer Cell. (2010) 18:74–87. 10.1016/j.ccr.2010.04.025 20609354 PMC2904512

[35] Dutta D Barr VA Akpan I Mittelstadt PR Singha LI Samelson LE. Recruitment of calcineurin to the TCR positively regulates T cell activation. Nat Immunol. (2017) 18:196–204. 10.1038/ni.3640 27941787 PMC6352896

[36] Fric J Lim CXF Koh EGL Hofmann B Chen JM Tay HS. Calcineurin/NFAT signalling inhibits myeloid haematopoiesis. EMBO Mol Med. (2012) 4:269–82. 10.1002/emmm.201100207 22311511 PMC3376854

