# Supplementary figures and images for: Nfatc4 Deficiency Attenuates Ototoxicity by Suppressing Tnf-Mediated Hair Cell Apoptosis in the Mouse Cochlea
Source: Front Immunol. 2019 Jul 17;10:1660. doi: 10.3389/fimmu.2019.01660 (PMC6650568; doi:10.3389/fimmu.2019.01660)

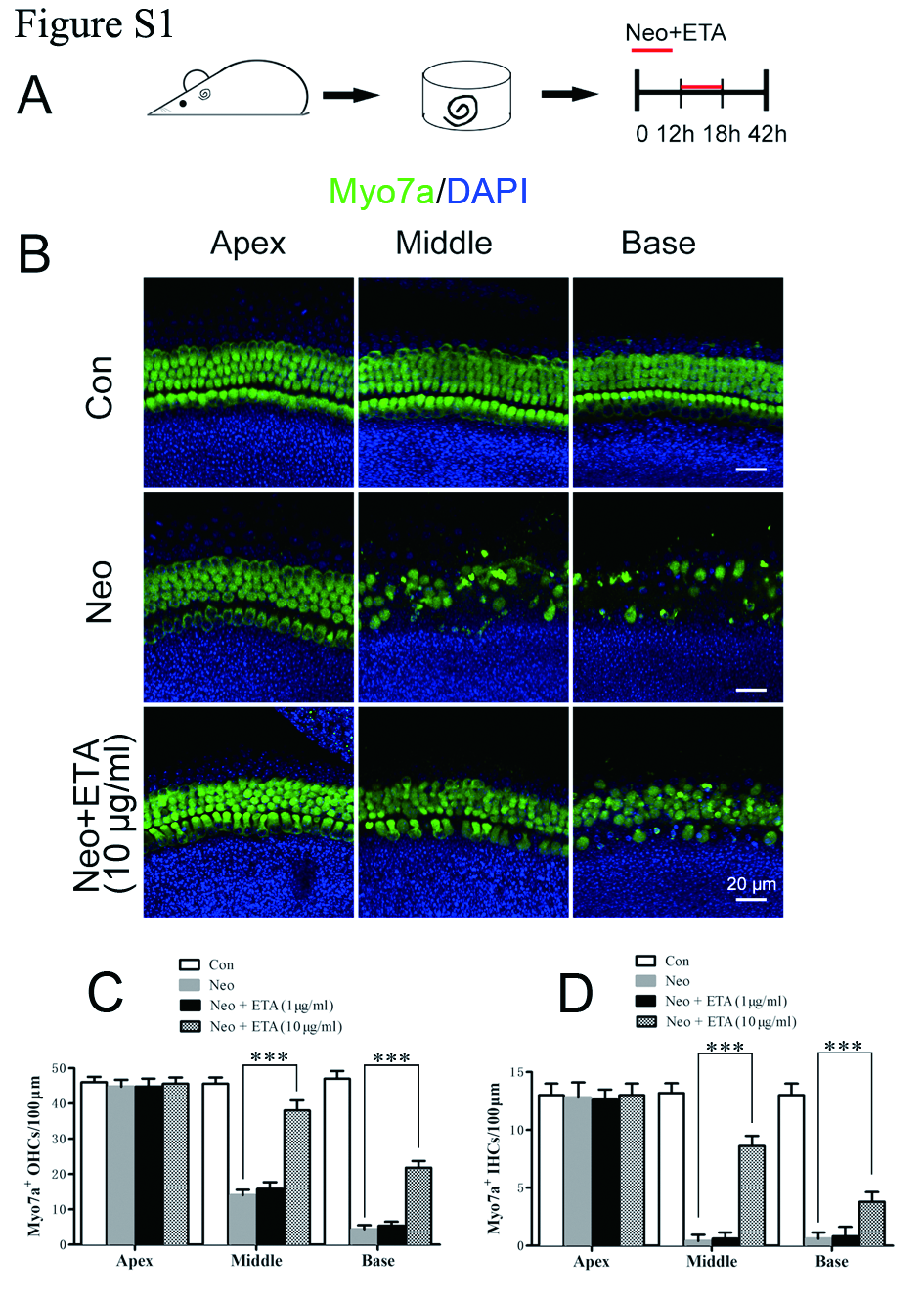

Supplement: Figure S1 — Etanercept treatment protected cochlear hair cells against neomycin-induced damage. (A) The diagram of the assay for (B–D). Cochlear sensory epithelium samples from P2 WT mice were dissected out and allowed to recover for 12 h. The samples were treated with 1 mM neomycin and etanercept (ETA, 1 μg/ml or 10 μg/ml) for 6 h, allowed to recover for 24 h, and then used for immunostaining. (B) The representative Myo7a immunofluorescence staining after drug treatment (neomycin alone or combined with ETA). (C,D) ETA treatment at 10 μg/ml protected against neomycin-induced hair cell loss. Scale bar = 20 μm. ***indicates p < 0.001. n = 5. [file Image_1.TIF]

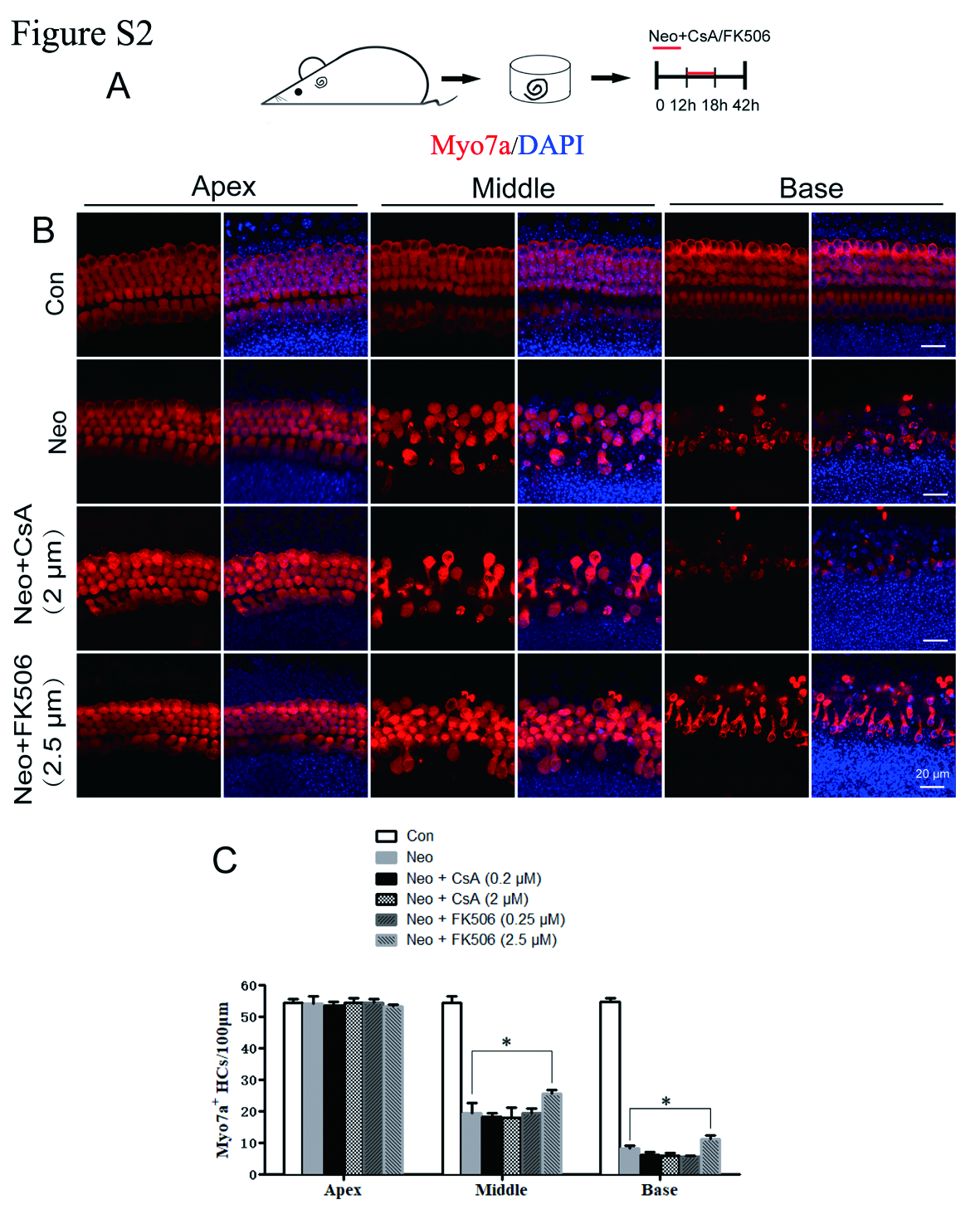

Supplement: Figure S2 — The effects of calcineurin/NFAT signaling inhibitors on the survival of cochlear hair cells against neomycin-induced damage. (A) The diagram of the assay for (B,C). Cochlear sensory epithelium samples from P2 WT mice were dissected out and allowed to recover for 12 h. The samples were treated with 1 mM neomycin and CsA/FK506 for 6 h, allowed to recover for 24 h, and then used for immunostaining. (B) The representative Myo7a immunofluorescence staining after drug treatment. (C) Quantification of the number of HCs. Scale bar = 20 μm. *indicates p < 0.05. n = 5. [file Image_2.TIF]

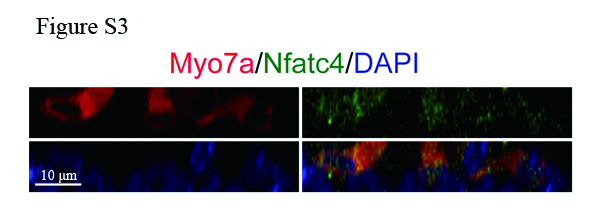

Supplement: Figure S3 — Immunofluorescence staining of Nfatc4 in neomycin combined with Len treated cochlear epithelium. Scale bar = 10 μm. [file Image_3.TIF]
